# Genome Sequence and Phylogenetic Analysis of the Sulfide-Oxidizing Heliobacterium “*Heliomicrobium sulfidophilum*” Strain BR4

**DOI:** 10.3390/microorganisms14051160

**Published:** 2026-05-21

**Authors:** John A. Kyndt, Kristina O. Petrova, Stepan V. Toshchakov, Irina A. Bryantseva

**Affiliations:** 1 College of Science and Technology, Bellevue University, Bellevue, NE 68005, USA; 2 Kurchatov Center for Genome Research, NRC “Kurchatov Institute”, Ac. Kurchatov Square, 1, 123098 Moscow, Russia; petrova.k.o@yandex.ru (K.O.P.);; 3 Winogradsky Institute of Microbiology, Research Center of Biotechnology, Russian Academy of Sciences, 33, Bld. 2 Leninsky Ave., 119071 Moscow, Russia; bryantseva@mail.ru

**Keywords:** *Heliomicrobium*, bacteriochlorophyll *g*, photosynthetic reaction center, whole genome sequence, heterodisulfide reductase

## Abstract

*Heliobacteraceae* are unique endospore-forming photosynthetic bacteria that are known for possessing the simplest photosynthetic apparatus of any known organism. More genomic and physiological analysis is needed to further understand the evolution of photosynthesis and the unique metabolic pathways of nitrogen and sulfur metabolism in this family. Here, we present the genome and phylogenetic analysis of “*Heliomicrobium sulfidophilum*” strain BR4^T^, which was isolated previously from an alkaline sulfide-containing hot spring. In addition to the presence of a Type I reaction center, genes for bacteriochlorophyll *g* synthesis and nitrogenase system, the genomic analysis also explains the need for biotin as a supplied growth factor in *Heliomicrobium* species. The *Heliobacteriaceae* genome comparison also revealed a previously unidentified gene cluster of heterodisulfide reductase-like proteins (Hdr genes) and molybdopterin-based enzymes for polysulfide reductase. The whole-genome comparison, including ANI, dDDH, and single-gene phylogenetic analyses, confirms the correct placement of strain BR4 in the *Heliomicrobium* genus and strengthens the overall phylogenetic distribution of the *Heliobacteriaceae*.

## 1. Introduction

Species of the family *Heliobacteriaceae* (called heliobacteria) are anoxygenic phototrophic bacteria that are phylogenetically and physiologically distinct from other species in this group (purple and green bacteria). Heliobacteria belong to the class *Clostridia*, the order *Eubacteriales* (formerly *Clostridiales*) of the phylum *Bacillota* (formerly *Firmicutes*) [1,2]. Currently, four genera (*Heliobacterium* (*Hbt*.), *Heliomicrobium* (*Hmb*.), *Heliophilum* (*Hph*.), and *Heliorestis* (*Hrs*.)) and eleven species are described in the family *Heliobacteriaceae* [3]. They are phylogenetically Gram-positive bacteria but are phenotypically Gram-negative due to their atypical cell wall structure (a thin peptidoglycan layer and the lack of an outer membrane [4]. All species of *Heliobacteriaceae* contain a unique photosynthetic pigment, bacteriochlorophyll (Bchl) *g*, a pigment absent in all other anoxygenic phototrophs [4,5]. In heliobacteria, photosynthetic pigments reside in the cytoplasmic membrane rather than in highly differentiated intracytoplasmic membranes typical of purple bacteria (vesicles and lamellae) or the chlorosomes of green bacteria [4,5]. Heliobacteria are obligate anaerobes, lack all known biochemical pathways for autotrophic growth, and produce heat-resistant endospores [6,7].

A heliobacterium strain BR4 was isolated from the cyanobacterial mat of an alkaline sulfide-containing hot Bol’sherechenskii spring (pH 9.3; H_2_S 10 mg/1; 50 °C) located in the Barguzinskii reserve, near the Bol’shaya River, 30 km away from Lake Baikal (Russia) in 1993, whereas before that time heliobacteria had been isolated only from paddy soils. The new isolate differed from other heliobacteria that were known at that time by a higher pH optimum of 7–8 and a relatively low temperature optimum (30 °C), and also tolerated high concentrations of hydrogen sulfide (up to 2 mM at pH 7.4) and oxidized it to elemental sulfur in the light and in the presence of organic compounds [8]. The strain BR4 was described as the type strain of a new species under the name “*Heliobacterium sulfidophilum*” in 2000 and included in the Validation List of IJSEM [4,9,10]. Later, “*Hbt. sulfidophilum*” was recognized as not validly published because the type strain BR4^T^ (=UNIQEM 113^T^ = UQM 40069^T^) is not deposited in at least two different recognized culture collections [11]. As a result of a systematic reconsideration of species and genus assignments of heliobacteria based on phenotypic properties, fatty acid composition, DNA–DNA hybridization data, and 16S rRNA comparisons, “*Hbt. sulfidophilum*” was included in the genus *Heliomicrobium* and renamed “*Heliomicrobium sulfidophilum*” [3]. However, at the time of that publication, there was no whole genome sequence available for “*Hmb. sulfidophilum*” to complete a full genomic comparison to the other sequenced *Heliomicrobium* species.

Currently, the genome sequences of eight of the eleven known species of heliobacteria have been determined and are available [3,12,13,14]. We now sequenced the genome sequence of “*Hmb. sulfidophilum*” to further analyze its similarities and unique differences with the other species of the family *Heliobacteriaceae*. This article presents the genome sequence and genetic and phylogenetic analysis of the “*Hmb. sulfidophilum*” strain BR4^T^.

## 2. Materials and Methods

### 2.1. DNA Extraction

Genomic DNA was prepared from cells frozen in liquid nitrogen using the QIAGEN MagAttract HMW DNA Kit (Qiagen, Hilden, Germany) according to the manufacturer’s protocol for Gram-positive bacterial cultures. The quality of extracted DNA was assessed spectrophotometrically using a Nanodrop 1000 spectrophotometer (Thermo Fisher Scientific, Waltham, MA, USA) by measuring the absorbance ratios at A260/A280 and A260/A230. DNA concentration was determined using a Qubit 4.0 fluorometer (Thermo Fisher Scientific, Waltham, MA, USA) with the Qubit dsDNA HS Assay Kit (Thermo Fisher Scientific, Waltham, MA, USA).

### 2.2. Genome Sequencing

For long-read sequencing, unsheared genomic DNA was used for library preparation with the Ligation Sequencing Kit (SQK-LSK109, Oxford Nanopore Technologies, Oxford, UK) with barcoding performed using the Native Barcoding Expansion kit (EXP-NBD104, Oxford Nanopore Technologies, UK), according to the manufacturer’s instructions. Sequencing of the samples was performed on a GridION sequencer using FLO-MIN106D flow cells (Oxford Nanopore Technologies, UK). Basecalling and demultiplexing procedures were performed by MinKNOW using Guppy v.6.5.7 and the “FLO-MIN106 DNA—High-Accuracy” model for SQK-LSK109. This generated 93.13 Mbps sequencing data (14,191 reads) with an average read length of 6567 bp.

For the short-read sequencing libraries, the genomic DNA was fragmented with Covaris^TM^ M220 (Woburn, MA, USA) Focused-ultrasonicator to a mean fragment size of 500 bp. The libraries were prepared with NebNext^TM^ Ultra II DNA library preparation kit (New England Biolabs, Ipswich, MA, USA) using manufacturer’s instructions. The libraries were sequenced with SurfSeq 5000^TM^ sequencer (GeneMind, Shenzhen, China) using 2 × 150 bp paired-end sequencing reagents. This generated 683.18 Mbp of high-quality short-read sequencing data after adapter trimming.

### 2.3. Genome Assembly and Annotation

An assembly of the short read Surfseq data alone, using Unicycler (v0.4.8) in BV-BRC [15,16], provided a high-quality assembly, but with a relatively high number of contigs (145 contigs). Parameters for the Unicyler assembly were: unicycler -t 12 -o . --min_fasta_length 300 --keep 2 --no_pilon; min_contig_coverage_threshold: 5.0 min_contig_length_threshold: 300. Polishing was performed with two rounds of pilon. The assembly of the Nanopore data alone, using flye (v2.9.1-b1780) in BV-BRC [15,17], resulted in fewer contigs (9 in total), however, of lower quality and more assembly repeats (coarse consistency 98.7%; fine consistency 89.0%). Parameters for the fly assembly were: flye --out-dir . --genome-size 5,000,000 --threads 12; min_contig_coverage_threshold: 5.0, min_contig_length_threshold: 300. Polishing was performed using two rounds of Racon. Therefore, we used the Nanopore ligation assembly as a scaffold for the Surfseq data and performed a Minimap2 aligned assembly in Geneious Prime (v2026.0.1) (minimap2_macos -x sr --frag=yes --secondary=yes -N 5 -p 0.8 -a refSeq.fasta input.fasta -o output.sam) [18]. In total, 4,567,272 of the 4,571,321 short reads were assembled. This provided the best result with a high-quality assembly into 5 contigs and an accumulative 228× coverage. The coarse and fine consistency were improved to 99.3% and 98.6%. The final assembled genome was 100% complete according to CheckM [19]. A Mobile Element Detection analysis was performed using the geNomad pipeline in BV-BRC [20]

The final assembly was annotated using the Rastk toolkit annotation in BV-BRC [15,21]. Parameters used: “taxonomy_id”: “2831443”, “lowvan_min_contig_length”: 300, “code”: 0, “lowvan_max_contig_length”: 35,000, “analyze_quality”: “1”, “queue_nowait”: “1”. This resulted in 3439 CDS and 104 tRNAs.

### 2.4. Phylogenetic Analysis

A whole-genome-based phylogenetic tree with the *Heliobacteriaceae* genomes, including the strain BR4 genome, was generated using the CodonTree method within BV-BRC [15], which uses PGFams as homology groups. The following genomes were used in the analysis for comparison: *Heliobacterium mobile* DSM 6151^T^, *Heliobacterium chlorum* DSM 3682^T^, *Heliomicrobium undosum* DSM 13378^T^, *Heliomicrobium gestii* DSM 11169^T^, *Heliomicrobium modesticaldum* DSM 9504^T^, *Heliorestis convoluta* DSM 19787^T^, *Heliorestis acidaminivorans* DSM 24790^T^, *Heliophilum fasciatum* DSM 11170^T^. *Dehalobacter* sp. CF was used as an outgroup [22]. A total of 584 PGFams were found among these selected genomes using the CodonTree analysis, and the aligned proteins and coding DNA from single-copy genes were used for RAxML analysis (v 8.2.12) [23,24], which uses 100 rounds of “Rapid bootstrapping (RaxML -# option)” to generate support values for whole-genome phylogenetic trees. RAxML parameters were: raxmlHPC-PTHREADS-SSE3 -m GTRCAT -p 12345 -T 12 -f a -x 12345 -N 100. iTOL was used for tree visualization [25].

Average percentage nucleotide identity (ANIb) between the whole genomes was calculated using JSpecies [26], using default parameters. Digital DNA–DNA Hybridization (dDDH) data was obtained using the Type (Strain) Genome Server (TYGS) web server (https://tygs.dsmz.de accessed on 19 February 2026) [27]. The program used the distance formula d4 to calculate a similarity based on sequence identity. 16S rRNA and SQR and HdrA protein sequence identity comparisons were performed using LALIGN at EMBL-EBI [28].

The multiple sequence alignments for the BchG and reaction center Type1 comparisons were performed using MUSCLE [29]. The evolutionary history was inferred by using the Maximum Likelihood method and Whelan and Goldman + Freq. model [30]. The percentage of trees in which the associated taxa clustered together is shown next to the branches. Initial tree(s) for the heuristic search were obtained automatically by applying Neighbor-Join and BioNJ algorithms to a matrix of pairwise distances estimated using the JTT model, and then selecting the topology with superior log-likelihood value. A discrete Gamma distribution was used to model evolutionary rate differences among sites (5 categories (+G, parameter = 0.6163) for BchG and 5 categories (+G, parameter = 2.0529) for the reaction center tree). The trees are drawn to scale, with branch lengths measured in the number of substitutions per site. The BchG analysis involved 9 amino acid sequences, with a total of 310 positions in the final dataset. The reaction center analysis involved 10 amino acid sequences with a total of 786 positions in the final dataset. For both analyses, the protein sequences were obtained from the genome sequences used in the WGS tree. The *Chlorobaculum* sp. 24CR P480 reaction center was used as an outgroup for the PSI tree [31], while the BchG tree was midpoint-rooted. Evolutionary analyses were conducted in MEGA 11 [32,33], and iTOL was used for tree visualization [25].

For synteny analysis, comparative genome regions were generated in BV-BRC using global PGFam families to determine a set of genes that match a focus gene [15]. All *Heliobacteriaceae* genomes were used in the search and compared with the BR4 genome. The gene set is compared with the focus gene using BLAST (v2.17.0) and sorted by BLAST scores within BV-BRC. Heterodisulfide reductase A (HdrA) was used as focus gene to analyze synteny of the gene cluster. Conserved protein domain searches were performed using the NCBI CD-Search website (https://www.ncbi.nlm.nih.gov/Structure/cdd/wrpsb.cgi; last accessed 1 May 2026).

## 3. Results

### 3.1. Genome Properties

**Figure 1:**
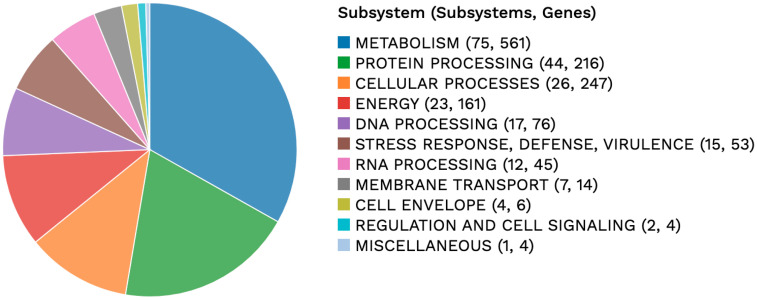
Overview of the subsystems and associated genes identified in “*Heliomicrobium sulfidophilum*” BR4 genome. The number of subsystems and associated genes identified are listed in parentheses.

The genome of strain BR4 was assembled into five contigs with a total genome size of 3,403,727 bp and a GC percentage of 57.2%, with an N50 of 2,286,869 bp. The genome was sequenced with a 228× coverage and was 100% complete according to CheckM [19]. A Mobile Element Detection analysis using the geNomad pipeline in BV-BRC did not find any evidence for any of the contigs being an isolated plasmid. Instead, several phage elements appear to be integrated in the contigs as prophages, as further discussed below. A total of 3439 CDS were identified after annotation, of which 2077 were proteins with a functional assignment (Table S1). A single set of rRNA sequences and 3 CRISPR arrays with 138 repeats were identified. A subsystem is a set of proteins that together implement a specific biological process or structural complex [34] assigned during BV-BRC annotation. An overview of the subsystems for the BR4 genome is provided in Figure 1. A total of 941 of the annotated proteins had subsystem assignments, with the majority involved in metabolism, protein processing, cellular and energy processes (Figure 1).

A comparison of genome characteristics to the other currently sequenced *Heliobacterium*/*Heliomicrobium*/*Heliophilum*/*Heliorestis* genomes is in Table 1. The genome size and GC content is within the expected range for this group. A JSpecies comparison of bidirectional average nucleotide identity (ANIb) comparison shows that the BR4 strain genome has 82.7–85.5% ANI with *Hmb. gestii*, *Hmb. modesticaldum*, and *Hmb. undosum*, which is similar to what these have to each other (Table 1) [3]. The ANI values with *Heliobacterium*, *Heliorestis,* and *Heliophilum* species are lower (66.3 to 71.8%; Table 1). All these are substantially below the arbitrary species cutoff of 95% [26], confirming that “*Hmb. sulfidophilum*” BR4 is correctly placed as its own unique taxonomic species.

A pairwise digital DNA–DNA hybridization (dDDH) analysis also showed the *Heliomicrobium* species to be closest to the new BR4 genome (between 27.0 and 31.1%), and the other genera more distantly related, with the exception of *Hrs. convoluta*, which showed a similar dDDH (27.1%) to the *Hmb. gestii* value (27.0%) (Table 1; https://tygs.dsmz.de accessed on 19 February 2026).

**Figure 2:**
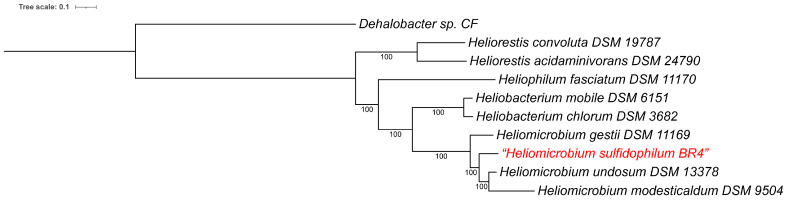
Whole-genome-based phylogenetic tree of the *Heliobacteriaceae.* The support values for the phylogenetic tree are generated using 100 rounds of the ‘Rapid bootstrapping’ option of RaxML. The branch length tree scale is defined as the mean number of substitutions per site, which is an average across both nucleotide and amino acid changes. All strains of heliobacteria on the tree are type strains. *Dehalobacter* sp. CF was included in the tree as an outgroup [22]. The new genome for strain BR4 is indicated in red. iTOL was used to draw the phylogenetic tree [25].

A whole-genome-based phylogenetic analysis showed that the strain BR4 clusters with the other three *Heliomicrobium* genome sequences (Figure 2), which form a separate clade from the other genera. This aligns well with the phylogenetic analysis using 16S rRNA sequences that was performed earlier [3]. The genome-derived 16S rRNA sequence from strain BR4 is 98.4% identical (98.9% similar, in 1514 nt overlap) to the “*Hmb. sulfidophilum*” 16S rRNA sequence that was deposited to Genbank earlier (AF249678 and NR_025090.1). The nearest homolog, *Hmb. undosum*, shows only 97.0% identity (97.5% similar, in 1499 nt overlap) with the genome-derived 16S rRNA sequence, indicating that the BR4 sequence does indeed belong to “*Hmb. sulfidophilum*”. The differences with the earlier 16S rRNA sequence are likely PCR-induced or sequencing errors in the earlier sequence, as several of the differences were at uncertain positions (N and R) or were repetitions of neighboring bases in the earlier sequence.

The ANI, dDDH, and WGS-based phylogenetic analysis all confirm that the earlier placement of “*Hmb. sulfidophilum*” within the *Heliomicrobium* genus was correct and strengthens the phylogenetic branch of this new genus.

The Comparative Systems analysis in BV-BRC allows for a pangenomic comparison of protein families, pathways, and subsystems in selected genomes. We compared the “*Hmb. sulfidophilum*” BR4 genome with the other eight heliobacterial genomes and found it to have 88 unique PGFams; however, 55 of those belong to uncharacterized hypothetical proteins, and at least 10 of these belong to mobile element or phage-related genes.

### 3.2. Central Carbon Metabolism

All heliobacteria catabolize pyruvate as a preferred carbon source; however, depending on the species, pyruvate is fermented with or without the production of H_2_ [35]. In case of H_2_ production, this is catalyzed by pyruvate:ferredoxin oxidoreductase, or in case of no H_2_ production, using a pyruvate–formate lyase. The genome of BR4 contains a pyruvate–formate lyase (EC 2.3.1.54) gene but also contains genes for pyruvate:ferredoxin oxidoreductase (alpha, beta, and gamma subunits) (EC 1.2.7.1). The latter genes are only found in two other heliobacterial species: *Hmb. gestii* and *Hph. fasciatum.* The molecular basis of pyruvate fermentation in *Heliobacteriaceae* has not been studied in more detail, but based on the genomic differences, it may be diverse among the different heliobacterial species.

The “*Hmb. sulfidophilum*” BR4 genome also contains genes for L-lactate permease and L-lactate dehydrogenase (EC 1.1.1.27), which allows it to use lactate as a carbon source. There is some difference among the heliobacteria when it comes to the metabolism of propionate. While “*Hmb. sulfidophilum*” was not able to be cultivated on propionate [9], species of *Hrs. acidaminivorans*, *Hrs. daurensis, Hmb. undosum*, and *Hrs. convoluta* have been able to perform propionate photoassimilation [5,36]. Genes for propionyl-CoA carboxylase, methylmalonyl-CoA epimerase, and methylmalonyl-CoA mutase, which catalyze the conversion of propionyl-CoA to succinyl-CoA before entering the citric acid cycle, are all present in the “*Hmb. sulfidophilum*” BR4 genome; however, a propionyl-CoA carboxylase that catalyzes the first step in propionate assimilation was not found. In *Hrs. convoluta*, it was suggested that an encoded methylmalonyl-CoA carboxyltransferase could circumvent such deficiency [13]. However, we did confirm the presence of the same methylmalonyl-CoA carboxyltransferase (EC 6.4.1.2) in the *“Hmb. sulfidophilum*” BR4 genome. Therefore, the differences in propionate assimilation observed between the various heliobacterial species appear to have a more complex underlying biochemical mechanism.

As in other *Heliobacteriaceae*, except *Hph. fasciatum* [14], anaplerotic CO_2_ assimilation occurs through the activity of a phosphoenolpyruvate (PEP) carboxykinase-like protein. A gene coding for PEP carboxykinase-like protein was found in the BR4 genome (PGF_07340469). All genes for a complete citric acid cycle were found in strain BR4, as in all other *Heliobacteriaceae*, including the Re face-specific citrate synthase (EC 2.3.3.3), which is a specific type of citrate synthase found primarily in strictly anaerobic bacteria and archaea that uses the Re-face of oxaloacetate [37].

Like other heliobacteria, “*Hmb. sulfidophilum*” BR4 is incapable of photoautotrophic growth and lacks genes for any of the Calvin-Benson cycle. In addition, “*Hmb. sulfidophilum*” BR4 apparently also lacks a gene for citrate lyase, similar to other heliobacteria, making it incapable of autotrophic growth using the reverse citric acid cycle as seen in green sulfur bacteria [38].

### 3.3. Photosynthetic Reaction Center and BchG Analysis

**Figure 3:**
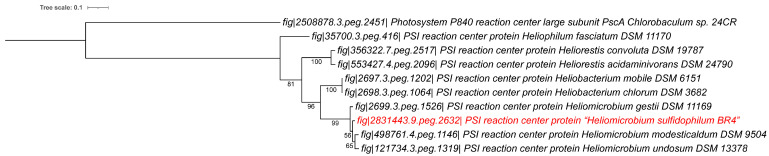
Phylogenetic tree of the *Heliobacteriaceae* using the type I reaction center protein sequences. Sequences were translated sequences derived from the whole genome sequences. Accession numbers are indicated for each sequence. The phylogenetic tree was calculated in MEGA11 using the Maximum Likelihood method and Whelan and Goldman +Freq model (WAG+F), with Gamma distribution and allowing for some sites to be evolutionarily invariable. Bootstrap values were generated from 500 bootstrapping rounds. All strains of heliobacteria on the tree are type strains. *Chlorobaculum* sp. 24CR P480 reaction center was used as an outgroup for the PSI tree [31]. The new strain BR4 sequence is indicated in red. iTOL was used to draw the phylogenetic tree [25].

All species of the family *Heliobacteriaceae* have unique Type I photosynthetic reaction centers that appear to have a very ancient evolutionary separation from the other Type I reaction centers of *Chlorobiota* (formerly *Chlorobi*), *Chloracidobacteriaceae* (called chloracidobacteria), and *Cyanobacteriota* (formerly *Cyanobacteria*) (the latter have both a Type I and Type II reaction center) [39]. The homodimeric Type I photosynthetic reaction centers from heliobacteria, *Chlorobiota,* and chloracidobacteria have presumably directly evolved from a primordial homodimeric photosynthetic ancestral system [39]. Given the importance of photosynthesis and the conserved nature of these reaction center proteins, we performed a phylogenetic analysis of the heliobacterial PS Type I (PSI) protein sequences. As can be seen in Figure 3, the overall topology and the placement of the strain BR4 is very similar to those from the WGS-based analysis, where the four *Heliomicrobium* strains form a distinct clade separated from the other genera.

**Figure 4:**
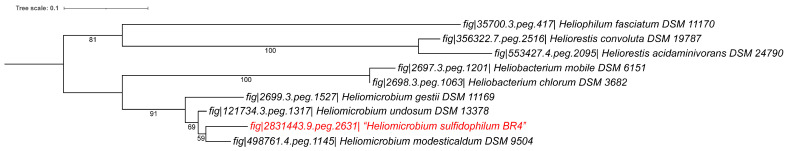
Phylogenetic tree of the *Heliobacteriaceae* using the BchG protein sequences. Sequences were translated from the whole genome sequences. Accession numbers are indicated for each sequence. The phylogenetic tree was calculated in MEGA11 using the Maximum Likelihood method and Whelan and Goldman +Freq model (WAG+F), with Gamma distribution and allowing for some sites to be evolutionarily invariable, and iTOL was used to draw the phylogenetic tree [25]. Bootstrap values were generated from 500 bootstrapping rounds. The tree was midpoint rooted, and the “*Heliomicrobium sulfidophilum*” BR4 protein sequence is indicated in red. All strains of heliobacteria on the tree are type strains.

Another unique feature of heliobacteria is that they contain Bchl *g* as the primary photosynthetic pigment, functioning as both the main antenna pigment and the special-pair electron donor (P798) [40,41]. Heliobacteria lack peripheral light-harvesting complexes, making Bchl *g* unique in directly absorbing light and transferring energy within their specialized, simplified reaction center complex. Bacteriochlorophyll *g* synthase (BchG) is an essential enzyme in heliobacteria that catalyzes the final step in the biosynthesis of Bchl *g*. We searched for and identified the bacteriochlorophyll *g* synthase (*bch*G) gene sequence in all of the sequenced genomes and generated a protein sequence-based phylogenetic tree with this unique protein as well. Figure 4 shows that this single protein tree has the same phylogenetic topology as found earlier for the PSI and whole genome comparisons.

### 3.4. CooS Analysis

In the earlier heliobacterial study, it was found that there are significant differences in the carbon monoxide metabolic enzymes that are encoded in their genomes [3]. Carbon monoxide is metabolized using the enzyme carbon monoxide dehydrogenase (CODH). Different protein families for carbon monoxide dehydrogenase were differentially found in heliobacterial genomes [3]. *Hmb. gestii, Hmb. undosum* and *Hph. fasciatum* were found to contain a less conventional CODH (CooS_2) and had a gene cluster surrounding that was unique to these three genomes. This was surprisingly missing from *Hmb. Modesticaldum*, and at that time, it was not clear if there was a physiological implication of this or if this was a technical consequence of the earlier assembly and annotation techniques used for that genome completion. We searched the new “*Hmb. sulfidophilum*” BR4 genome for the presence of carbon monoxide dehydrogenase genes and surrounding gene clusters, and found that, similar to *Hmb. modesticaldum* genome, the “*Hmb. sulfidophilum*” BR4 genome is also lacking the CooS carbon monoxide dehydrogenase and surrounding genes. This similarity between the two genomes suggests that the earlier observation in *Hmb. modesticaldum* was not an anomaly but indicates that there is indeed a different evolutionary history and possibly differences in carbon monoxide metabolism in these different species groups. A more detailed description of the CooS genes and synteny can be found in [3]; however, at this point, it is unclear if these missing genes in the two genomes have physiological implications since carbon monoxide metabolism in heliobacaterial species has not been studied yet.

### 3.5. Nitrogen Metabolism: Nitrogen Fixation

It was previously shown that all heliobacterial species contain the nitrogen fixation genes *nif*HDK, hydrogen uptake genes *hup*SLB, and the *nrf*AH genes for reduction of nitrite to ammonia [3]. The six genes encoding nifHDK (dinitrogenase and dinitrogenase reductase) and nifENB (nitrogenase assembly and maturase proteins) are present in the “*Hmb. sulfidophilum*” strain BR4, and the synteny of the *nif* gene cluster (*nif*I1-*nif*I2-*nif*H-*nif*D-*nif*K-*nif*E-*nif*N-*nif*X-*fdx*B-*nif*B-*nif*V) is conserved compared with the other sequenced *Heliomicrobium, Heliobacterium,* and *Heliorestis* genomes. This gene cluster has been shown to be essential and sufficient to express an active nitrogenase system [42]. Its presence in “*Hmb. sulfidophilum*” is consistent with the observation that strain BR4 can utilize molecular nitrogen as its nitrogen source [9].

### 3.6. Biotin Metabolism

It was previously observed that strain BR4 requires biotin as an essential growth factor [9], which is also true for most of the other heliobacteria, with the exception of *Heliorestis* species. The two *Heliorestis* genomes contain a complete biotin biosynthesis cluster: *bio*B-*bio*D-*bio*F-*bio*H-*bio*C-*bio*A. However, the BR4 genome contains the genes *bio*D-*bio*A, while *bio*B is located elsewhere in the genome, and appears to lack the *bio*F-*bio*H-*bio*C genes. This is similar to the other *Heliomicrobium* genomes, but different from *Hph. fasciatum,* which contains *bio*B, but lacks all the other genes from the *bio* gene cluster [14]. A KEGG pathway overview of biotin metabolism, with the present/absent genes in BR4 and their respective roles and EC numbers, can be found in Supplemental Figure S1. The *bio*F-*bio*H-*bio*C genes code for 8-amino-7-oxononanoate synthase (EC 2.3.1.47), biotin synthesis protein, and malonyl-[acyl-carrier protein] O-methyltransferase (EC 2.1.1.197), respectively, which are all essential enzymes in the biotin synthesis pathway, and their absence explains the need for biotin as a supplied growth factor in *Heliomicrobium* species. BioC (EC 2.1.1.197) is a key enzyme in the early steps of biotin biosynthesis in bacteria, and catalyzes the conversion of malonyl-acyl carrier protein (ACP) to malonyl-ACP methyl ester, using S-adenosyl-L-methionine (SAM) as the methyl donor (Figure S1), while BioF (EC 2.3.1.47) catalyzes the first committed step in biotin biosynthesis (Figure S1). It performs the decarboxylative condensation of L-alanine and pimeloyl-[acyl-carrier protein] to produce 8-amino-7-oxononanoate (AON), a critical step for assembling the fused heterocyclic rings of biotin. BioH is a crucial (alpha/beta)-hydrolase enzyme that catalyzes the hydrolysis of pimeloyl-ACP methyl ester to produce pimeloyl-ACP [43,44]. Although BioH is essential in most bacteria for biotin synthesis, in some species it can be replaced by the functional analog BioG, which performs the same esterase activity. A search for BioG also showed it to be absent from the strain BR4 genome (Figure S1). The absence of these essential genes for biotin synthesis in *Heliomicrobium* species is likely the cause of their need for biotin supplementation.

### 3.7. Sulfur Metabolism: Sulfide Oxidation

**Figure 5:**
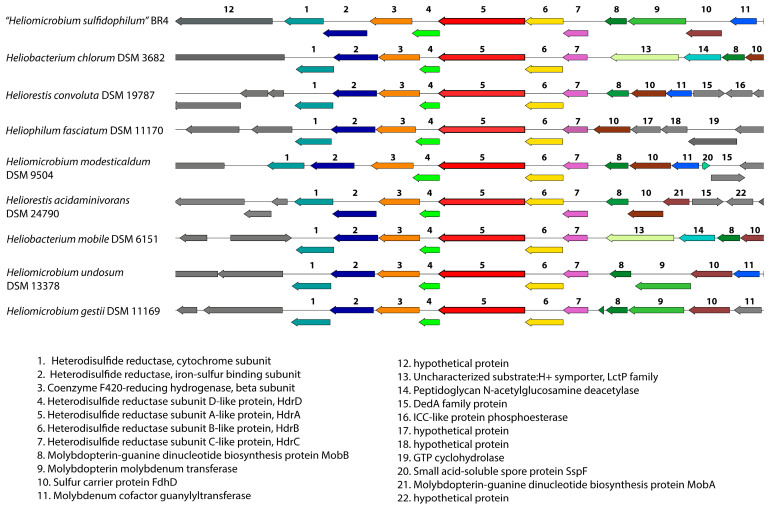
Synteny of the Hdr genomic region in *Heliobacteriaceae*. Synteny plots were generated in BV-BRC, which uses the Proteome Comparison tool. Genes are colored based on their family membership. Only genes relevant to the Heterodisulfide reductase and molybdopterin-based enzymes for polysulfide reductase activity are labeled for clarity.

Since strain BR4 was originally described as one of the first heliobacterial species that was able to oxidize sulfide and tolerate high concentrations of sulfide [8,9] we searched the genome for the following enzymes that are typically important for sulfide/sulfur oxidation in anaerobic bacteria: sulfide:quinone oxidoreductase (SQR); flavocytochrome c-sulfide dehydrogenase (FccAB); dissimilatory sulfite reductase, and heterodisulfide reductase (Hdr). We searched both the annotated genes and also performed additional BLAST searches with these enzymes from *Chlorobaculum* species. None of these proteins have been characterized in *Clostridia*, and there are no closely related reference proteins for these types of enzymes, which is why we used the more distant *Chlorobaculum* homologs for these searches. None of these genes appear to have homologs in strain BR4 or in any of the other heliobacterial genomes, except for homologs of the heterodisulfide reductase-like proteins (Hdr genes). Hdr enzymes and their pathways have originally been studied mainly in archaeal methane metabolizing species, but more recently homologs have been found in a wider range of bacteria [45,46], although their presence in heliobacteria has never been described before. There are some variations in the components and operon layout (called types or modules), but these Hdr enzymes all play roles in methanogenesis, sulfate reduction, and lithotrophic reduced sulfur compound oxidation [45,46,47,48]. When searching the heliobacterial genomes for these gene clusters, we found that all of them have the HdrABC and a smaller HdrD component, and also have the upstream molybdopterin-based enzymes for polysulfide reductase activity, in addition to the downstream heterodisulfide cytochrome reductase, iron–sulfur binding protein, and hydrogenase. Figure 5 shows a synteny plot of the entire *hdr* and the surrounding gene region in the heliobacterial genomes. The heterodisulfide gene synteny is well conserved in all of these genomes, but there is some variation in the upstream molybdopterin-based enzymes for polysulfide reductase. The latter could relate to some of the differences observed in sulfide metabolism in the different species; however, this will require further analysis.

**Figure 6:**
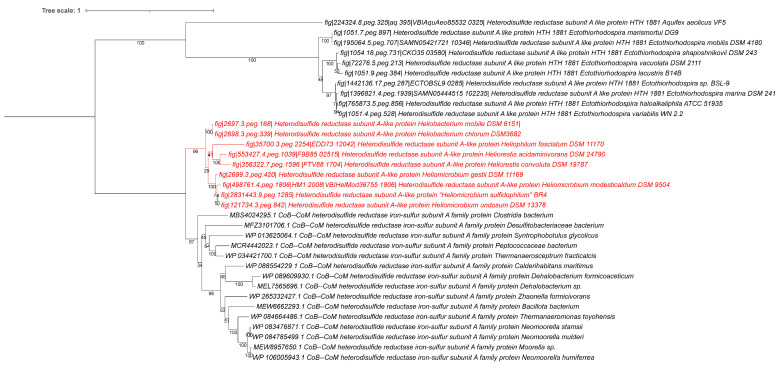
Phylogenetic tree of the *Heliobacteriaceae* HdrA and closely related protein sequences. Accession numbers from the whole genome sequences or NCBI Genbank submissions are indicated for each sequence. The phylogenetic tree was calculated in MEGA11 [32,33] using the Maximum Likelihood method and Le_Gascuel_2008 model. A discrete Gamma distribution was used to model evolutionary rate differences among sites (5 categories (+G, parameter = 1.0518)). The rate variation model allowed for some sites to be evolutionarily invariable ([+I], 10.99% sites). This analysis involved 34 amino acid sequences. There were a total of 669 positions in the final dataset. iTOL was used to draw the phylogenetic tree [25]. Bootstrap values were generated from 500 bootstrapping rounds. The tree was midpoint rooted, and *Heliobacteriaceae* HdrA protein sequences are indicated in red. All strains of heliobacteria on the tree are type strains.

The closest homologs of the HdrA protein (from “*Hmb. sulfidophilum*” BR4) is found in MAG genomes from *Peptococcaceae* bacteria and *Thermanaerosceptrum fracticalcis* (also a *Peptococcaceae* bacterium), but only with 70–72% identity. A broader phylogenetic analysis that included homologous HdrA proteins from related families such as the *Ectothiorhodospiraceae* and *Aquificaceae* showed that those Hdr proteins are more distantly related (Figure 6). An LALIGN analysis between the “*Hmb. sulfidophilum*” BR4 HdrA protein and the ones from *Aquifex* and *Ectothiorhodospira* species showed lower identities of only 37–39%. The more distant HdrA subunits are also significantly shorter than the heliobacterial ones and belong to a different class (about 350 aa vs. 660 aa). A conserved domain analysis also shows that the heliobacterial HdrA proteins have the entire conserved ‘hetero-SS-HdrA2 domain’ for a CoB-CoM heterodisulfide reductase HdrA2, commonly found in archaeal methanogens. A recent study of HdrA subunits in *Archaea* shows that there is substantial domain and structural variation in the HdrA subunit, and four major types can be distinguished [45]. The heliobacterial HdrA is most similar to the type II system that resulted from a fusion of two different HdrA types (hence the larger size). The archaeal type II HdrA is part of a gene cluster containing molybdopterin oxidoreductase and F420 dehydrogenase, similar to what we observed in the heliobacterial Hdr synteny.

These Hdr proteins are not well studied outside the archaeal methanogenic species, and their function in heliobacteria remains speculative at this point. The most likely candidates for the single initial step of sulfide to sulfur oxidation are SQR and FccAB [49]; however, no homologs of these were found in any of the heliobacterial genomes sequenced thus far. Since Hdr-like systems have been shown to play roles in lithotrophic reduced sulfur compound oxidation, for example, in *Aquifex* and *Hyphomicrobium denitrificans* [50,51], and given the apparent absence of SQR and FccAB genes, one could speculate about a possible role of the Hdr-like system in the observed sulfur oxidation of “*Hmb. sulfidophilum*”; however, these Hdr systems belonged to different types and had different gene clusters. Therefore, without further biochemical and physiological analysis, the specific role of the Hdr genes in sulfur metabolism in heliobacterium remains enigmatic.

When searching for SQR in other *Clostridia*, we found it annotated in several uncultured *Clostridia* or MAGs from *Clostridia*-like species. When using that putative clostridial SQR gene, it identified a gene (PGF_00024531, annotated as ‘NAD dehydrogenase’) in the heliobacterial species as the only homolog. This gene is most similar to the Type VI and Type I SQR proteins of other purple and green sulfur bacteria, but only with 28.0% protein identity (61.0% similarity to *Chlorobium tepidum* TLS). The heliobacterial protein contains an Ndh domain; however, both bacterial SQR and NAD dehydrogenase are membrane-associated proteins that share a similar structural fold (belonging to the two-Dinucleotide Binding Domains Flavoprotein tDBDF superfamily), although they are distinct enzyme families. In addition, the heliobacterial protein does have the key functional SQR amino acids conserved, as indicated in [49], including the catalytic cysteine residues and the capping loop 1 conserved glutamate residue. However, given the very low homology, further biochemical and proteomics analysis will be needed to identify whether this gene indeed codes for a distant heliobacterial SQR protein.

In addition, “*Hmb. sulfidophilum*” also lacks the sulfur-oxidizing *sox* genes, as do all other known heliobacteria. These genes encode enzymes necessary to oxidize thiosulfate, which is a reduced inorganic sulfur compound commonly oxidized as an electron donor by anoxygenic phototrophs. The lack of these genes is consistent with the fact that *Heliobacteriaceae* do not oxidize thiosulfate.

### 3.8. Phage Regions

It is noteworthy to mention that two of the five contigs of the strain BR4 genome consist nearly entirely of phage-related, mobile elements, and CRISPR-related genes. Based on the geNomad mobile element detection analysis, these phage elements appear to be integrated in the contigs as prophages, and there is no indication that these are circular, isolated plasmids. Contig 2 has a size of 109,597 bp and a total of 157 CDS (98 are hypothetical proteins) and 14 CRISPR regions, while contig 3 has a smaller size of 34,397 bp and 48 CDS, with only five annotated phage-related genes and the rest hypothetical proteins. Phage-related genomic regions are not unique to the “*Hmb. sulfidophilum*” BR4 genome and can be found in all other heliobacterial genomes as well; however, the closely related *Hmb. gestii* and *Hmb. undosum* appear to have much smaller phage regions (only 7–9 phage-related genes) as compared with “*Hmb. sulfidophilum*” and *Hmb. modesticaldum*, which could indicate historically more recent phage encounters by the latter two *Heliomicrobium* species.

## 4. Conclusions

Several of the findings from the genomic analysis help explain the ecological role of “*Hmb. sulfidophilum*” BR4 as it serves as a specialized recycler in its habitat, specifically the cyanobacterial mats of an alkaline sulfide-containing hot spring.

As a photoheterotroph, it sustains itself by consuming organic acids like pyruvate, lactate, and butyrate in the presence of light [9]. The new genome sequence analysis provides the genetic insight for the metabolism of these nutrients but also opens up opportunities for further differentiating studies between the various heliobacterial species. For example, the presence of multiple genes for pyruvate fermentation, and the presence of homologous genes in species that have different capabilities for propionate assimilation, trigger the need for further biochemical studies.

Like other heliobacteria, “*Hmb. sulfidophilum*” is a diazotroph, providing valuable nitrogen input to its, possibly nutrient-limited, anaerobic environment. We confirmed the presence of the necessary nitrogenase gene cluster and its conservation amongst heliobacteria. Their ability to fix atmospheric nitrogen likely contributes to nutrient boosts to its habitat.

Unlike many other phototrophs, “*Hmb. sulfidophilum*” is highly sulfide-tolerant, playing a niche role in the sulfur cycle by oxidizing sulfide into elemental sulfur [8,9]. Although we were unable to identify homologs of the most likely candidates for the single initial step of sulfide to sulfur oxidation (SQR and FccAB), we did identify a gene cluster for heterodisulfide reductase (Hdr) enzymes and the upstream molybdopterin-based enzymes for polysulfide reductase activity, in addition to the downstream heterodisulfide cytochrome reductase, iron–sulfur binding protein, and hydrogenase. These were previously thought to be primary features of methane-producing *Archaea* and had never been described in heliobacteria. While the Hdr gene arrangement is well conserved across different heliobacteria, the specific upstream enzymes vary, which might explain why different species handle sulfide differently. Although this needs further biochemical and physiological confirmation studies, it is intriguing to speculate that *Heliobacteriaceae* use a specialized, *Archaea*-like system for sulfur oxidation, which supports the sulfur cycle in their anaerobic environments.

Beyond its sulfur processing and nitrogen fixation, the metabolism of *Heliobactriaceae* is uniquely resilient because of their capability of forming heat-resistant endospores, ensuring survival during environmental shifts or desiccation [3,6,12,13,14]. When sunlight is unavailable, many heliobacterial species, including “*Hmb. sulfidophilum*”, can pivot from light-driven energy production to a slow fermentation of pyruvate [7,9]. This metabolic flexibility, combined with their status as distant relatives in the *Clostridia*, makes them an interesting bridge between ancient anaerobic fermenters and more complex photosynthetic life.

The analysis of the complete genome sequence of “*Hmb. sulfidophilum*” strain BR4 revealed commonalities with other heliobacteria (lack of autotrophy, a Type I reaction center, genes for bacteriochlorophyll *g* synthesis and nitrogenase system, etc.) and provided further insight into the metabolism of heliobacteria (e.g., the sulfur metabolism, the requirement for biotin as a growth factor). A comparative whole-genome analysis, including ANI, dDDH, and phylogenetic analysis of single genes, confirmed the correct placement of strain BR4 to the genus *Heliomicrobium*. Currently, the status of the species “*Hmb. sulfidophilum*” has not yet changed.

## 5. Emended Description of “*Heliomicrobium sulfidophilum*” Kyndt et al. 2021

*Heliomicrobium sulfidophilum* (sul.fi.do’phi.lum. N.L. neut. n. *sulfidum* sulfide; N.L. masc. adj. *philus* (from Gr. masc. adj. *philos*) liking; N.L. neut. adj. *sulfidophilum* liking sulfide).

Basonym: *Heliobacterium sulfidophilum* Bryantseva et al. 2001.

The description is as given for *Heliobacterium sulfidophilum* [9].

Type strain: BR4^T^, UNIQEM 113^T^, UQM 40069^T^.

The DNA G+C content as calculated from the genome sequence of strain BR4^T^ is 57.2 mol%, while the G+C content determined by thermal denaturation is 51.3 mol%.

The full genome DNA sequence for the strain BR4^T^ is registered under accession no. JBVTZZ000000000 under BioProject PRJNA1429406 and BioSample SAMN56011572. The genome size is 3.4 Mb.

The 16S rRNA gene sequence of the strain BR4^T^ is registered at GenBank under accession no. AF249678 and NR_025090.1. The genome-derived 16S rRNA gene sequence is registered at NCBI GenBank under accession number PZ240011.

## Figures and Tables

**Table 1:** Genome features of the “*Heliomicrobium sulfidophilum*” BR4^T^ and other species of the *Heliobacteriaceae*.

Species	Genome Size	GC Content	Contigs	Coverage	N50	CDS	tRNAs	ANIb (%) *	dDDH (%) *	Reference	Genbank Accession #
“*Hmb. sulfidophilum*” BR4^T^	3.4 Mb	57.2	5	228×	2,286,869	3439	104	−	−	this paper	JBVTZZ000000000
*Hmb. undosum* DSM 13378^T^	3.8 Mb	57.1	71	59×	176,377	3784	104	85.4	30.9	[3]	WXEY00000000
*Hmb. gestii* DSM 11169^T^	3.7 Mb	57.2	54	50×	284,497	3593	90	82.7	27	[3]	WXEX00000000
*Hmb. modesticaldum* DSM 9504^T^	3.1 Mb	56	1	NA	NA	2701	109	85.5	31.1	[12]	NC_010337
*Hbt. chlorum* DSM 3682^T^	4.1 Mb	48.7	214	15×	130,004	4379	153	71.8	19.8	[3]	JACVHF000000000
*Hbt. mobile* DSM 6151^T^	4.1 Mb	49.1	151	57×	123,143	4179	154	71.8	19.8	[3]	WNKU01000000
*Hrs. acidaminivorans* DSM 24790^T^	3.0 Mb	41	44	36×	281,451	2941	75	66.3	19.9	[3]	WBXO01000000
*Hrs. convoluta* DSM 19787^T^	3.2 Mb	43.1	1	NA	NA	2908	104	66.7	27.1	[13]	NZ_CP045875
*Hph. fasciatum* DSM 11170^T^	3.1 Mb	50.9	73	NA	NA	3111	90	68.6	20.9	[3]	SLXT01000000

* Values relative to “*Hmb. sulfidophilum*” BR4^T^. NA, no data available.

## Data Availability

This Whole Genome Shotgun project has been deposited at DDBJ/ENA/GenBank under BioProject PRJNA1429406 and BioSample SAMN56011572. The whole genome accession number is JBVTZZ000000000. The version described in this paper is version JBVTZZ010000000. The raw reads have been submitted to the Sequence Read Archive (SRA) database with the following accession numbers: SRR37487494 (SurfSeq dataset) and SRR37487553 (Oxford Nanopore datasets). The 16S rRNA gene sequence was submitted to NCBI GenBank under accession number PZ240011.

## Supplementary Materials

The following supporting information can be downloaded at: https://www.mdpi.com/article/10.3390/microorganisms14051160/s1, Table S1: Overview of the assembly and annotation scores and detailed genome characteristics of the “*Hmb. sulfidophilum*” BR4 genome. Assembly and annotations were performed in BV-BRC. Figure S1: KEGG pathway overview of the biotin metabolism pathway. The enzymes involved in biotin synthesis for which the genes are annotated in the “*Hmb. sulfidophilum”* BR4 genome are marked in green boxes. Missing genes in the biotin synthesis pathway are in white boxes.

## Abbreviations

The following abbreviations are used in this manuscript:

Bchl	Bacteriochlorophyll
WGS	Whole genome sequence
ANI	Average nucleotide identity
ANIb	Bidirectional average nucleotide identity
dDDH	Digital DNA–DNA hybridization
PS	Photosynthetic system
SQR	Sulfide:quinone oxidoreductase
Hdr	Heterodisulfide reductase
CDS	Coding Sequence
