# Supplementary material for: Genome Sequence and Phylogenetic Analysis of the Sulfide-Oxidizing Heliobacterium “Heliomicrobium sulfidophilum” Strain BR4
Source: Microorganisms. 2026 May 21;14(5):1160. doi: 10.3390/microorganisms14051160 (PMC13209566; doi:10.3390/microorganisms14051160)
Supplement: Supplementary file 1 [file microorganisms-14-01160-s001.zip › TableS1_BR4_genomedetail_editable.pdf]

Genome Name	***Heliomicrobium sulfidophilum* BR4**
Coarse consistency (%)	99.3
Fine consistency (%)	98.6
Completeness (%)	100
Contamination (%)	0
Contig count	5
DNA size (bp)	3403727
GC Content	57.187195
Contigs N50 (bp)	2286869
Contigs L50	1
Overpresent Roles	12
Underpresent Roles	7
Predicted Roles	1357
Completeness Roles	24
Total Distinct Roles	1826
% Protein-Encoding Feature Coverage	101.04
CDS	3,439
CDS Ratio	1.0103631
Hypothetical CDS	1362
Hypothetical CDS Ratio	0.467287
Repeat Regions	49
tRNA	104
rRNA	3
Partial CDS	0
Hypothetical proteins	1,362
Proteins with functional assignments	2,077
Proteins with EC number assignments	709
Proteins with GO assignments	592
Proteins with Pathway assignments	523
Proteins with Subsystem assignments	941
crispr_repeat	138
crispr_spacer	135
crispr_array	3
Antibiotic Resistance (PATRIC)	22
Antibiotic Resistance (CARD)	6
