# Supplementary figures and images for: Genome Sequence and Phylogenetic Analysis of the Sulfide-Oxidizing Heliobacterium “Heliomicrobium sulfidophilum” Strain BR4
Source: Microorganisms. 2026 May 21;14(5):1160. doi: 10.3390/microorganisms14051160 (PMC13209566; doi:10.3390/microorganisms14051160)

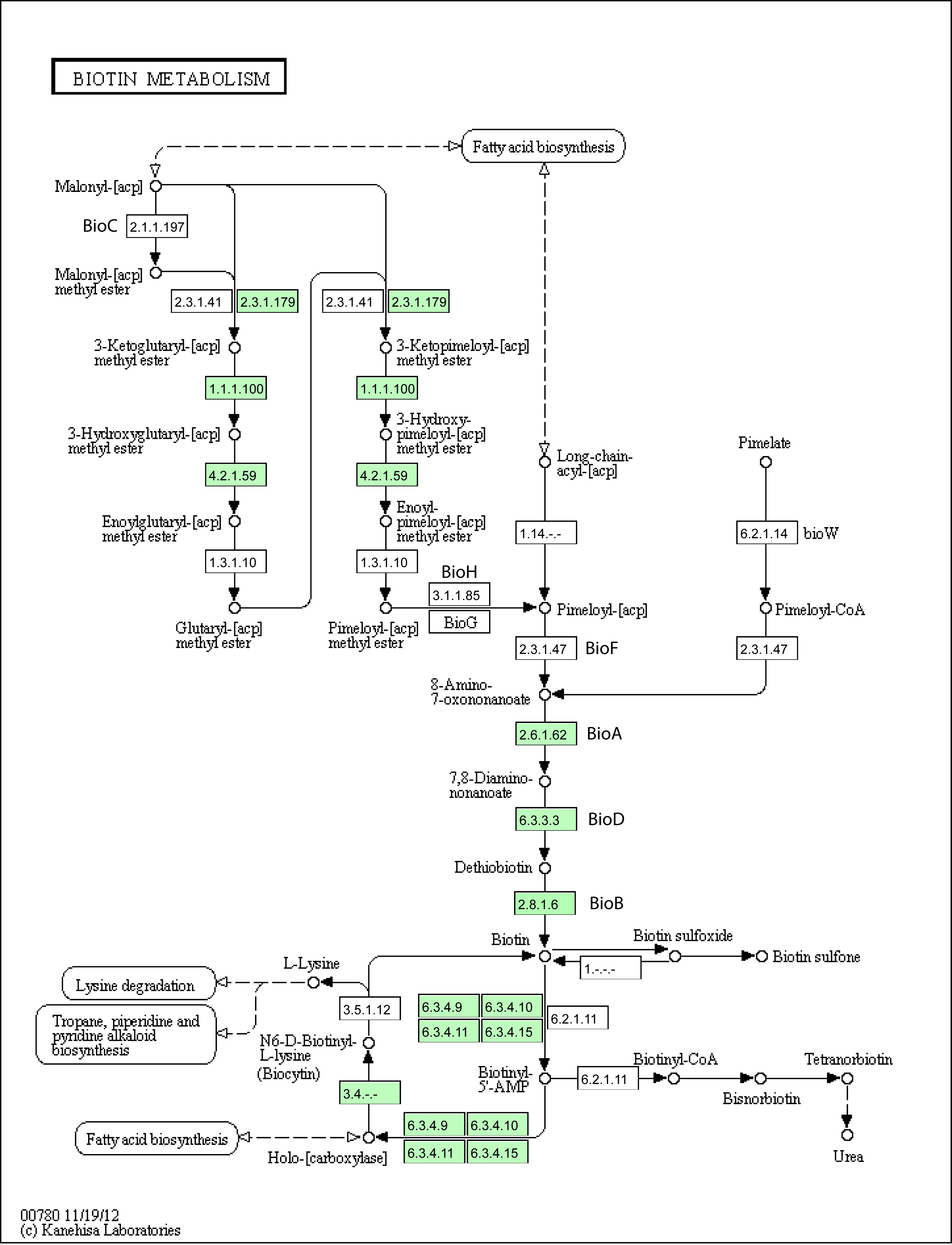

Supplement: Supplementary file 1 [file microorganisms-14-01160-s001.zip › FigS1_Biotin KEGGmap_BR4_BVBRC_pathway_map.tif]
